# Supplementary material for: Asynchronous magnetic resonance elastography: Shear wave speed reconstruction using noise correlation of incoherent waves
Source: Magn Reson Med. 2022 Oct 27;89(3):990–1001. doi: 10.1002/mrm.29502 (PMC9792433; doi:10.1002/mrm.29502)
Supplement: Supplementary file 1 — DATA S1 MATLAB code used to generate the numerical simulations of Figure 1 [file MRM-89-990-s002.zip › k-Wave/helpfiles/example_pr_2D_tr_circular_sensor.html]

2D Time Reversal Reconstruction For A Circular Sensor Example (k-Wave)


# 2D Time Reversal Reconstruction For A Circular Sensor Example

This example demonstrates the use of k-Wave for the time-reversal reconstruction of a two-dimensional photoacoustic wave-field recorded over a circular array of sensor elements. The sensor data is simulated and then time-reversed using `kspaceFirstOrder2D`. It builds on the 2D Time Reversal Reconstruction For A Line Sensor Example.

- Open the file in the MATLAB Editor
- Run the file in MATLAB

## Contents

- Simulating the sensor data
- Avoiding the inverse crime
- Interpolating incomplete sensor data

## Simulating the sensor data

The sensor data is simulated using `kspaceFirstOrder2D` with an external image map for the initial pressure distribution in the same way as the Loading External Image Maps Example (this time the image is representative of vasculature). A plot of the initial pressure distribution (scaled to 256 by 256 grid points) and the Cartesian sensor mask used to record the pressure field (70 sensor points over a 270 degree measurement angle) is shown below.


## Avoiding the inverse crime

To avoid the inverse crime (in which computations are run forwards and backwards using the same parameters and discretisation thereby possibly disguising any underlying errors), in this example the time-reversal reconstruction is computed using measurement data with added noise (using `addNoise`) and a different computational grid. The use of a different reconstruction grid size is straightforward for a Cartesian sensor mask; in the forward simulation the pressure time-series at the Cartesian points are computed using interpolation and can thus be transposed to a grid of any size. The time-reversal reconstruction using a computational grid with 300 by 300 grid points is shown below.


## Interpolating incomplete sensor data

In the reconstruction shown above, the edges of original image have been significantly blurred. This is due to outgoing waves from each discrete detector position on the measurement surface interacting with other positions at which a pressure value is also being enforced during the time reversal. This interaction can be avoided by interpolating the recorded data onto a continuous (rather than discrete) measurement surface within the k-space grid used for the reconstruction. This can be achieved using `interpCartData` and a binary sensor mask of a continuous arc that is spatially equivalent to the original Cartesian measurement surface. This function calculates the equivalent time-series at the sensor positions on the binary sensor mask from those on the Cartesian sensor mask via interpolation (nearest neighbour is used by default).

```
% create a binary sensor mask of an equivalent continuous circle
sensor_radius_grid_points = round(sensor_radius / kgrid_recon.dx);
binary_sensor_mask = makeCircle(kgrid_recon.Nx, kgrid_recon.Ny, kgrid_recon.Nx/2 + 1, kgrid_recon.Ny/2 + 1, sensor_radius_grid_points, sensor_angle);

% assign to sensor structure
sensor.mask = binary_sensor_mask;

% interpolate data to remove the gaps and assign to sensor structure
sensor.time_reversal_boundary_data = interpCartData(kgrid_recon, sensor_data, cart_sensor_mask, binary_sensor_mask);
```

Details of the interpolation are printed to the command line.

```
Interpolating Cartesian sensor data...
  interpolation mode: nearest
  number of Cartesian sensor points: 70
  number of binary sensor points: 574
  computation completed in 0.013681s
```

The reconstructed image and a profile through x = -0.5 are shown below. The interpolation has considerably sharpened the edges of the reconstruction and the magnitude has also been improved through partial correction for the discontinuous aperture.
